# Modulatory effect of filarial infection on the systemic hormone levels in subjects with metabolic syndrome (DM-LF5)

**DOI:** 10.3389/fendo.2022.1011942

**Published:** 2022-11-22

**Authors:** Joy Manohar Sibi, Viswanathan Mohan, Mohan Deepa, Subash Babu, Vivekanandhan Aravindhan

**Affiliations:** ^1^ Department of Genetics, Dr. A. L. Mudaliar Post Graduate Institute of Basic Medical Sciences, University of Madras, Chennai, India; ^2^ Madras Diabetes Research Foundation and Dr. Mohan’s Diabetes Specialties Centre, ICMR Center for Advanced Research on Diabetes and IDF Centre of Excellence in Diabetes Care, Chennai, India; ^3^ National Institute of Health-International Centre for Excellence in Research, National Institute for Research in Tuberculosis, Chennai, India

**Keywords:** metabolic syndrome, filariasis, incretins, adipokines, insulin, glucagon

## Abstract

**Aim:**

Metabolic syndrome (MS) refers to a group of co-morbidities which include central obesity, hypertension, hyperglycemia and dyslipidemia. Previously, we reported that childhood lymphatic filariasis (LF) confers significant protection against type-1 and type-2 forms of diabetes, by means of immunomodulation. In the present study, we studied the effect of LF on endocrine dysfunction in MS and Non-MS patients in baseline and after 10 years of follow-up.

**Methods:**

We quantified the serum levels of pancreatic hormones (insulin and glucagon), incretins (Ghrelin, GIP and GLP-1) and adipokines (leptin, adiponectin, adipsin, visfatin, PAI-1 and resistin) by multiplex bead array system.

**Results:**

MS (both LF- and LF+) subjects had increased insulin levels compared to NMS (both LF- and LF+) subjects. MS-LF+ subjects had significantly increased levels of glucagon, ghrelin, GIP and GLP-1 and decreased levels of adipsin, compared to MS-LF- subjects. Interestingly this effect was short-lived and was not seen in the follow-up samples.

**Conclusion:**

Overall, LF infection might confer limited short-term beneficial effects against MS, by means of modulating the incretin levels,either directly or indirectly.

## Introduction

Metabolic syndrome (MS) refers to a group of co-morbidities which include central obesity, hypertension, hyperglycemia and dyslipidemia (1). Patients with MS have an increased risk of developing cardiovascular, cerebral and hepatic disorders (1). MS being a metabolic disorder is associated with three types of hormonal dysregulation, namely: 1. Pancreatic hormones, 2. Incretins and 3. Adipokines. Insulin and glucagon are the major pancreatic hormones which control blood glucose levels, lipid storage and protein synthesis (2). Incretins are a group of low molecular weight peptides secreted by the intestine which control insulin secretion and action (3). Ghrelin, Gastric Inhibitory Peptide (GIP) and Glucagon-Like Peptide-1 (GLP-1) are the major incretins implicated in obesity and diabetes (3). Finally, adipokines are a group of peptides secreted by the adipose tissue and control various aspects of metabolism (4). Adiponectin, adipsin, leptin, resistin, visfatin and Plasminogen Activator Inhibitor (PAI)-1 are the major adipokines which control insulin secretion and action (4). Recently, a systematic meta-analysis of fourteen studies showed that fasting blood glucose and HbA1c were significantly lower in persons with helminth infection, and the prevalence of metabolic syndrome and type-2 diabetes was lower among those who were infected (5).

Helminth infections affect approximately one-quarter of the world’s population and are widespread in lower to middle-income countries (6). The prevalence of MS is predominant in urbanized countries where most helminth infections have been eliminated (7, 8). Recent data in both animal and human studies have shown a reciprocal relationship between helminth infection and metabolic disorders like obesity, insulin resistance and type-2 diabetes (9–13), suggesting that helminth infection may have a role in the prevention or delay of these diseases (metabolic hygiene hypothesis). In previous studies, we have shown that glycemic factors, hormone and cytokine levels in T2DM were modulated by *Strongyloides stercoralis* infection, which is partially reversed following anti-helmintic therapy (14). However, the mechanisms of how helminth infections mediate protection against MS is unknown.

In our previous studies, we found out that childhood lymphatic filariasis (LF) infection can confer significant protection against both type-1 (15) and type-2 diabetes (16), but not against the related coronary artery disease (17), largely by suppressing the metainflammation (18). However, the effect of LF in regulating endocrine dysfunction in these diseases is not known. Following our initial studies, several studies have reported the therapeutic utility of helminth products against metabolic disorders (7–9, 12, 13). Immunoendocrinology is a fast upcoming interdisciplinary field wherein several immunomodulatory agents were shown to either directly or indirectly alter hormone levels (11). Towards this end, in the present study, we have quantified the levels of pancreatic hormones, incretins and adipokines in MS and non-MS subjects (both LF infected and uninfected) cohort, at baseline and after 10 years of follow-up. The hormone levels were correlated with the clinical parameters and their association with the disease phenotype was studied.

## Materials and methods

### Study groups

The study participants were drawn from the Chennai Urban Rural Epidemiology Study (CURES), an epidemiological study conducted on the representative population of Chennai. Since the percentage of LF+ subjects among diabetes patients was just 4%, as reported previously (16), we had to screen 1416 subjects with various grades of glucose intolerance, to obtain the present sample size. Further, since the chronic diabetes subjects upon follow-up, had significant metabolic co-morbidities like hypertension, central obesity and dyslipidemia, we decided to study the effect of LF on MS, rather than isolated type-2 diabetes. The study subjects were thus divided into four groups: 1. Non-MS subjects who were LF^-^ (NMS-LF^–^ = 22); 2. Non-MS who were LF^+^ (NMS-LF^+^ = 22); 3. MS subjects who were LF^-^ (MS-LF^-^ = 21); 4. MS subjects who were LF^+^ (MS-LF^+^ = 23). MS was diagnosed as per the recommendation of the National Cholesterol Education Program (NCEP)–Adult Treatment Panel III criteria, modified for waist circumference, as per the WHO Asia Pacific guidelines (https://apps.who.int/iris/handle/10665/206936 **)** (10). MS was defined as the presence of any three of the following metabolic abnormalities: 1. Abdominal obesity (waist circumference ≥90cm for men; ≥ 80 cm for women); 2. High blood pressure (systolic blood pressure (SBP) ≥ 130 mm Hg or diastolic blood pressure (DBP) ≥ 85 mm Hg); 3. Elevated fasting blood glucose [FBG] ≥100 mg/dL; 4. Hypertriglyceridemia (Triglyceridies ≥150 mg/dL) or 5. Low HDL-Cholesterol (<40 mg/dL for men and <50 mg/dL for women). The study protocol was approved by the MDRF institutional ethical committee (Ref No-MDRF-EC/SOC/2009//05) and written informed consent was obtained from all the participants. The study was conducted as per the declaration of Helsinki, following STROBE guidelines. This is the 5^th^ manuscript from the DM-LF study.

### Inclusion and exclusion criteria

All study participants were recruited from the CURES study (16). None of the study subjects showed any symptoms of LF at the time of recruitment. The exclusion criteria were patients with type-1 diabetes and those with a previous diagnosis of urolithiasis, liver cirrhosis, congestive heart failure, chronic lung diseases, chronic infections or viral hepatitis, as reported previously (16).

### Anthropometry and biochemical measurements

Anthropometric measurements and biochemical parameters were measured as described previously (16).

### Detection of bancroftian LF

Serum filarial antigen levels were quantified using the *W. bancrofti* Og4C3 antigen-capture ELISA (Tropbio, Australia) according to the manufacturer’s instructions. A cut-off value of 128 antigen units was considered positive for LF (14).

### Estimation of pancreatic and gut hormones and adipokines

The serum levels of pancreatic hormones (insulin and glucagon), incretins (ghrelin, GIP and GLP-1) and adipokines (adiponectin, leptin, resistin, adipsin, PAI-1, visfatin) were estimated by Bio-Plex Pro Human Diabetes 10-Plex Assay and Bio-Plex Pro Human Diabetes Adipsin and Adiponectin Assays, as per the kit protocol (Biorad, USA). The lowest detection limits for the tested cytokines were: Insulin- 1.7 pg/ml, Glucagon- 15.7 pg/ml, Ghrelin- 8.01 pg/ml, GIP- 3.41 pg/ml; GLP-1- 5.91 pg/ml, Adiponectin- 160 pg/ml, Leptin- 11.5 pg/ml, Resistin-2.3pg/ml, Adipsin-43pg/ml, PAI-1- 14.24 pg/ml; Visfatin- 51.3 pg/ml.

### Statistical analyses

Mann-Whitney U test, Spearman’s correlation analysis, Person correlation analysis and multiple logistic regression analysis were performed using SPSS software. Multiple comparisons were corrected using Holm’s correction.

## Results

### Clinical characteristics of the study groups

**Table 1** shows the clinical characteristics of the study groups. Compared to NMS-LF^-^ subjects, NMS-LF^+^ subjects had increased waist circumference, BMI, TGL, HDL and LDL levels. MS-LF^+^ subjects had significantly increased total cholesterol and LDL levels compared to MS-LF^–^ subjects. Overall MS subjects had significantly increased age, waist circumference, BMI, SBP, DBP, FBS, PGBS, HBA1C, TGL and HDL levels compared to the NMS group. **Supplementary Table 1** shows the clinical characteristics of the study groups after follow-up. None of the LF+ subjects remained LF+, after 10 years of follow-up. Compared to baseline, the MS-LF- subjects had significantly increased fasting blood sugar levels in the follow-up. MS-LF+ subjects had significantly increased HDL levels in the follow-up study.

**Table 1:** Clinical characteristic of the study groups.

Clinical Parameters	NMS-LF–(n=22)		NMS-LF+ (n=22)	MS-LF- (n=21)	MS-LF+ (n=23)
**Age (years)**	35 ± 15.3		37 ± 16.8	46 ± 11.5**^b^	48 ± 11.01
**Sex (m/f)**	13M/9F		12M/10F	11M/10F	9M/14F
**TROPBIO**	10.9 ± 18.9		3544.4 ± 7105.1*^a^	8.9 ± 3.1	4414.7 ± 8744.1*^c^
**Waist (cm)**	70 ± 10.7		78 ± 9.4**^a^	96 ± 11.7***^b^	93 ± 11.7
**BMI (kg/m^2^)**	19 ± 3.4		22 ± 3.9*** ^a^	28 ± 5.2***^b^	25 ± 4.0
**Systolic BP (mmHg)**	110 ± 8.4		108 ± 13.1	140 ± 15.5***^b^	137 ± 21.1
**Diastolic BP(mm Hg)**	70 ± 9.1		70 ± 9.2	86 ± 10.2*** ^b^	83 ± 14.2
**FBS (mg/dl)**	82 ± 5.6		86 ± 7.0	113 ± 35.6**^b^	126 ± 44.2
**PGBS/PPBS**	101 ± 30.5		115 ± 36.7	191 ± 76.4***^b^	224 ± 107.3
**Glycated Haemoglobin (%)**	5 ± 0.5		6 ± 0.4	7 ± 1.5***^b^	7 ± 1.7
**Total Serum Cholesterol (mg/dl)**	167 ± 22.7		178 ± 34.5	189 ± 34.8*^b^	212 ± 40.0*^c^
**Serum triglycerides (mg/dl)**	71 ± 32.7		96 ± 36.4* ^a^	197 ± 6***^b^	185 ± 67.7
**HDL-cholesterol (mg/dl)**	51 ± 7.9		42 ± 8.1** ^a^	38 ± 6.8***^b^	41 ± 8.3
**LDL- cholesterol (mg/dl)**	101 ± 21.5		117 ± 31.6* ^a^	104 ± 40.0	134 ± 36.4** ^c^
**Microalbuminuria (mg/dl)**	9 ± 1.6		7 ± 4.9	15 ± 22.4	24 ± 34.9
**Urea**	21 ± 6.4		22 ± 5.3	20 ± 4.3	22 ± 5.1
**Creatinine**	1 ± 0.2		1 ± 0.9	1 ± 0.1	1 ± 0.2
**Diabetes status (%)**	NGT-95; PDM-5; NDM-0;KDM-0		NGT-77; PDM-23; NDM-0;KDM-0	NGT-14; PDM-62; NDM10; KDM-14	NGT-26; PDM-30; NDM-22;KDM-22
**Hypertension (%)**					
**Systolic BP**		0	5	76	65
**Diastolic BP**		5	5	76	43
**Central obesity (%)**		5	23	81	78
**Hypertriglyceridemia (%)**		5	9	81	70
**Dyslipidemia (%)**		9	64	90	83

BMI, Body Mass Index; FBS, Fasting Blood Sugar; PGBS, Post Glucose Blood Sugar/PPBS, Post Prandial Blood Sugar; HDL, High Density Lipoprotein; LDL, Low Density Lipoprotein.

^**a**^NMS-LF- vs NMS-LF+, ^**b**^MS-LF- vs NMS-LF+ and ^**c**^NMS-LF- vs MS-LF- groups were compared using Student t-test.

Data is shown as mean ± SD for continuous variables. Values highlighted in bold show significant difference.

* <0.05; ** <0.01; *** <0.001.

### Circulating levels of pancreatic and gut hormones in the MS-LF co-morbidity

**Figure 1:**
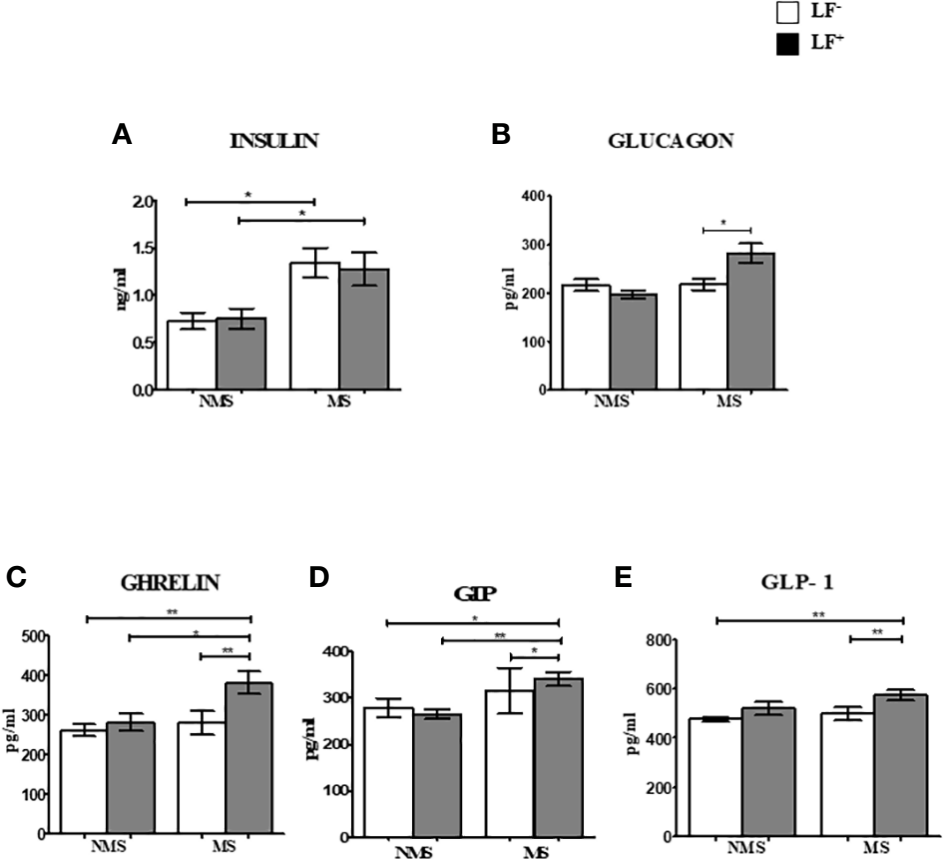
Baseline circulating levels of pancreatic and gut hormones in MS-LF study groups. Bar with error bars represent the mean ± SD of levels of insulin **(A)**, glucagon **(B)**, ghrelin **(C)**, GIP **(D)**, GLP-1 **(E)**, in the serum of the study subjects. The levels of pancreatic and gut hormones were estimated by Bio-Plex Pro Human Diabetes 10-Plex Assay. Statistical significance was determined by non-parametric Mann-Whitney U test and p<0.05 was considered significant. *p<0.05;**p<0.01.

**Figures 1A–E** shows the circulating baseline levels of pancreatic and gut hormones in the study groups. Insulin and glucagon levels were significantly increased in the MS groups (both LF^-^ and LF^+)^ compared to NMS-LF^–^ (control) and NMS-LF^+^ groups (**Figures 1A, B**). Ghrelin, GIP and GLP-1 levels were significantly increased in the MS-LF^+^ groups compared to NMS-LF^-^ and MS-LF^–^ groups (**Figures 1C–E**). **Supplementary Figures 1A–E** shows the circulating levels of pancreatic and gut hormones in the follow-up study groups. While significant differences were seen in the pancreatic and gut hormones at the baseline, these differences were nullified in the follow-up samples. It is important to note that none of the follow-up cases were LF+. Correlation analysis showed a positive correlation between glucagon levels with PGBS; and GIP-1 levels with urea, in the NMS-LF^–^ group (**Supplementary Table 2**). Ghrelin showed negative correlation with triglycerides; GIP showed positive correlation with FBS; GLP-1 showed negative correlation with PPBS and triglyceride, in the NMS-LF^+^ group (**Supplementary Table 3**). GIP showed negative correlation with SBP and positive correlation with HDL; GLP-1 showed positive correlation with DBP, in the MS-LF^–^ group (**Supplementary Table 4**). Glucagon showed positive correlation with FBS and PGBS; GIP showed positive correlation with triglycerides and creatinine, in the MS-LF^+^ group (**Supplementary Table 4**). Regression analysis showed a positive association between insulin levels with MS-LF^–^ and MS-LF^+^ groups, while glucagon, ghrelin and GIP showed positive association, only with the MS-LF^+^ group (**Supplementary Table 5**).

### Circulating levels of adipocytokines in the MS LF co-morbidity

**Figure 2:**
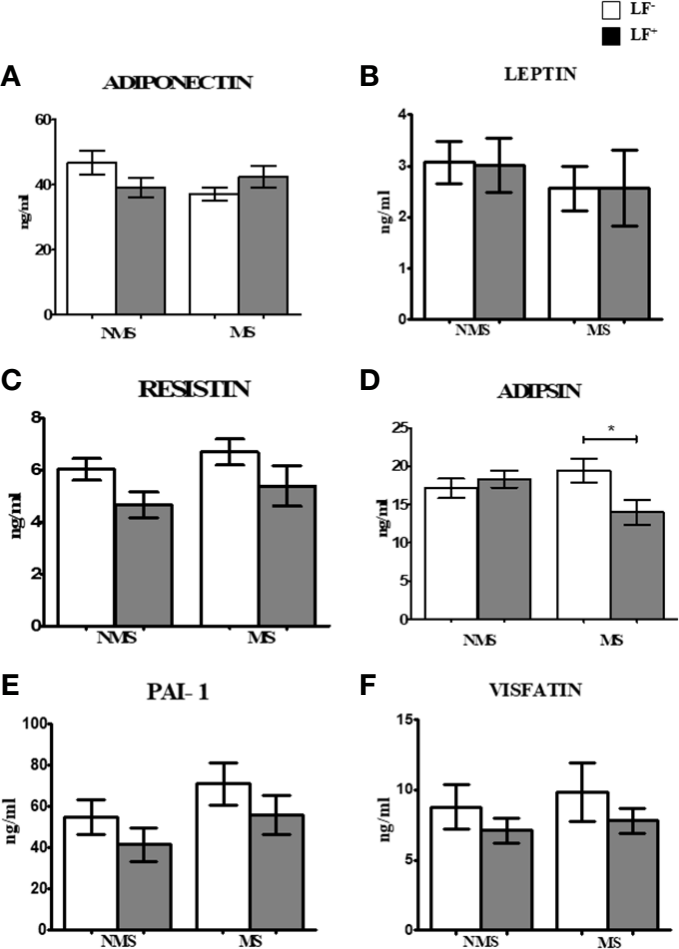
Baseline circulating levels of adipokines in MS-LF study groups. Bar with error bars represent the mean ± SD of levels of adiponectin **(A)**, leptin **(B)**, resistin **(C)**, adipsin **(D)**, PAI-1 **(E)** and visfatin **(F)** in the serum of study subjects. The levels of adiponectin and adipsin were estimated by Bio- Plex Pro Human Diabetes Adipsin and Adiponectin assay. Levels of leptin, resistin, PAI-1 and visfatin were estimated by Bio-Plex Pro Human Diabetes 10-Plex Assay. Statistical significance was determined by non- parametric Mann-Whitney U test and p<0.05 was considered significant. *p<0.05.

**Figures 2A–F** shows the circulating baseline levels of adipokines in the study groups. Adipsin was the only adipocytokine which was significantly reduced in MS-LF^+^ compared to MS-LF^-^ group. **Supplementary Figures 2A–F** shows the circulating levels of adipokines in the study groups after the follow-up. No significant difference was seen between the groups. In correlation analysis, adiponectin and resistin showed significant positive correlation with HDL; resistin showed negative correlation with waist circumference and creatinine, in the NMS-LF^-^ group (**Supplementary Table 2**). Adiponectin showed negative correlation with LDL; resistin and adipsin showed positive correlation with urea, in the NMS-LF^+^ group (**Supplementary Table 3**). Leptin showed positive correlation with HDL and urea, while resistin showed negative correlation with urea, in the MS-LF^-^ group (**Supplementary Table 4**). Leptin showed negative correlation with microalbuminuria. PAI-1 showed positive correlation with triglycerides and microalbuminuria and negative correlation with HbA1c, while visfatin showed positive correlation with creatinine, in the MS-LF^+^ group (**Supplementary Table 5**). In regression analysis, no significant association was seen between the adipocytokines and the study groups (**Supplementary Table 6**).

## Discussion

**Figure 3:**
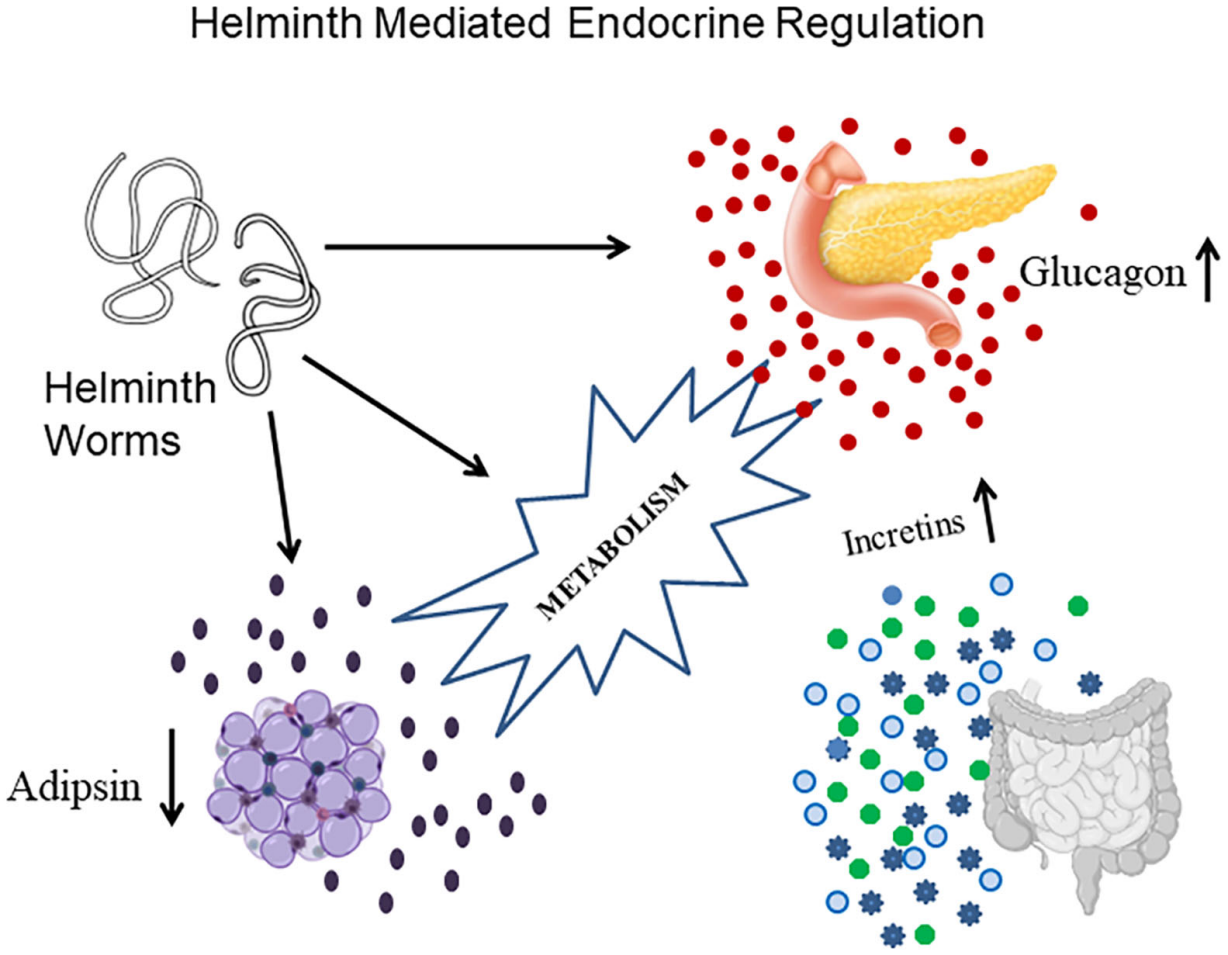
A model showing the complex endocrine regulation brought about by active helminth infection. Helminth infection increase the secretion of a) Glucagon from pancreas; b) Incretins from gut and c) Adipsin from adipose tissue. The beneficial effect of helminth infection in conferring protection against MS seems to be largely mediated through incretin secretion.

Previously, we have shown an immunomodulatory effect of LF infection on serum cytokine profile in type-2 diabetes patients (16). Recently, we have reported the effect of chronic diabetes on anti-filarial immunity (19). In the present paper, we have studied the effect of LF infection on serum hormone levels in MS patients. Our major findings were: 1. MS patients have significant insulin resistance, which is not modulated by LF infection; LF^+^ MS subjects had high glucagon levels compared to the LF^-^MS group. 2. LF infection increases incretin levels in MS subjects and 3. Adipsin levels were significantly increased in MS subjects who were LF^+^. The beneficial effect of LF infection, in reducing the morbidity associated with MS, seems to be, largely due to the upregulation of incretins, which increase both insulin secretion and decrease IR (**Figure 3**). However, this was a transient effect which was seen only during an active infection, since no long-term effect on serum hormone levels was noted in those, who have cleared the infection.

The serum levels of ghrelin, GLP-1 and GIP were found to be significantly elevated in the MS-LF^+^, compared to MS-LF^-^ group. The increased GIP levels (but not GIP-1 levels) were in accordance, with our recent study on obese subjects infected with *Strongyloides stercoralis* (20). The most unexpected finding of the study is that compared to pancreatic hormones and incretins, there was no major difference in the levels of the adipokines (except adipsin) between NMS-LF^-^ and MS-LF^-^ groups, which is in contrast to previous studies (21–26). In our previous study, we saw increased levels of adiponectin and decreased levels of resistin, visfatin, leptin and PAI-1 in obese individuals with *Strongyloides stercoralis* infection (20). Recently there had been renewed interest in understanding helminth-mediated endocrine regulation, which can confer protection against metabolic disorders (27). In fact, recent studies have indicated that the sudden drop in the prevalence of helminth infections, due to mass drug administration, may have fueled metabolic disorders in developing countries, which require urgent attention (28). In this respect, to the best of our knowledge, this is the first study to document filariasis-mediated modulation of hormonal levels in MS subjects.

The exact mechanism by which helminth infection modulates immunoendocrine functions is not clearly known. However, in a recent study in a high-fat diet-induced obese mice model, helminth *Strongyloides venezuelensis* infection, resulted in the alteration of gut microbiota, decreased gut permeability, decreased metabolic endotoxemia and decreased inflammation, which overall increased insulin sensitivity (29). Our study indicates that, apart from these effects, helminth infections might also induce the secretion of gut hormones, which adds one more level of complexity to the helminth-host interaction. The modulatory effect of LF on hormone profile was not seen after the follow-up period, within which all the infected individuals have cleared their infection. This observation is well in accordance with the outcome of a recent meta-analysis, which indicates that active helminth infection is more beneficial compared to past infection, since, constant taming of the immune system, by the worm, is needed in order to maintain metabolic homeostasis (5). In this connection, recently several novel helminth antigens with strong immunomodulatory effect have been identified. Mukherjee et al., have recently identified several filarial proteins which are capable of directly binding to TLR4 in macrophages (30, 31) and dendritic cells (32, 33). It is important to note that, apart from immune cells, adipocytes, hepatocytes, myocytes and pancreatic beta cells all express TLRs. However, whether the filarial products can directly bind to TLRs, present in these cells and modulate the cellular metabolism, is not known. While most of the helminth products induce metabolic homeostasis through immunomodulation, immunomodulatory glycan Lacto-N-fucopentaose III (LNFPIII), which is abundantly present in helminths, can directly bind to cell surface lectins present on hepatocytes and strongly suppress ectopic lipogenesis (34). By this mechanism, it can confer significant protection against steatohepatitis which is a major complication seen in MS (34).

The major limitations of this study are the limited sample size which could not be avoided due to the low prevalence of LF among the South Indian population. The major strength of the study is that, it was conducted in a filarial endemic population wherein the prevalence of MS is rapidly increasing. Further, all the important hormones which are implicated in MS have been quantified. Overall, LF infection offers some short-term benefits against MS, which needs further validation, in a large cohort study. To conclude, LF infection confers some immunoendocrine benefits against MS which seem to diminish, once the infection is cleared.

## Data availability statement

The raw data supporting the conclusions of this article will be made available by the authors, without undue reservation.

## Ethics statement

The studies involving human participants were reviewed and approved by MDRF institutional ethical committee. The patients/participants provided their written informed consent to participate in this study.

## Author contributions

VM, SB and VA conceived the idea. JS did the experiments. JS and VA did the analysis. MD was involved in sample collection and analysis. All authors contributed to the article and approved the submitted version.

## Funding

The Dept. of Genetics, University of Madras has received funds for infrastructural support from DST-FIST and UGC-SAP programs. The funders had no role in study design, data collection and analysis, decision to publish, or preparation of the manuscript.

## Conflict of interest

The authors declare that the research was conducted in the absence of any commercial or financial relationships that could be construed as a potential conflict of interest.

## Publisher’s note

All claims expressed in this article are solely those of the authors and do not necessarily represent those of their affiliated organizations, or those of the publisher, the editors and the reviewers. Any product that may be evaluated in this article, or claim that may be made by its manufacturer, is not guaranteed or endorsed by the publisher.

## Abbreviations

MS, Metabolic syndrome; LF, Lymphatic Filariasis; GIP, Gastric Inhibitory Polypeptide; GLP-1, Glucagon Like Peptide-1; PAI-1, Plasminogen Activator Inhibitor-1; FBS, Fasting Blood Sugar; PPBS, Post Prandial Blood Sugar; DBP, Diastolic Blood Pressure; SBP, Systolic Blood Pressure; TGL, Triglycerides; HDL, High Density Lipopolysaccharides; LDL, Low Density Lipopolysaccharides.
