# Supplementary material for: Modulatory effect of filarial infection on the systemic hormone levels in subjects with metabolic syndrome (DM-LF5)
Source: Front Endocrinol (Lausanne). 2022 Nov 22;13:1011942. doi: 10.3389/fendo.2022.1011942 (PMC9723321; doi:10.3389/fendo.2022.1011942)
Supplement: Supplementary file 1 [file Presentation_1.ppt]

## Slide 1
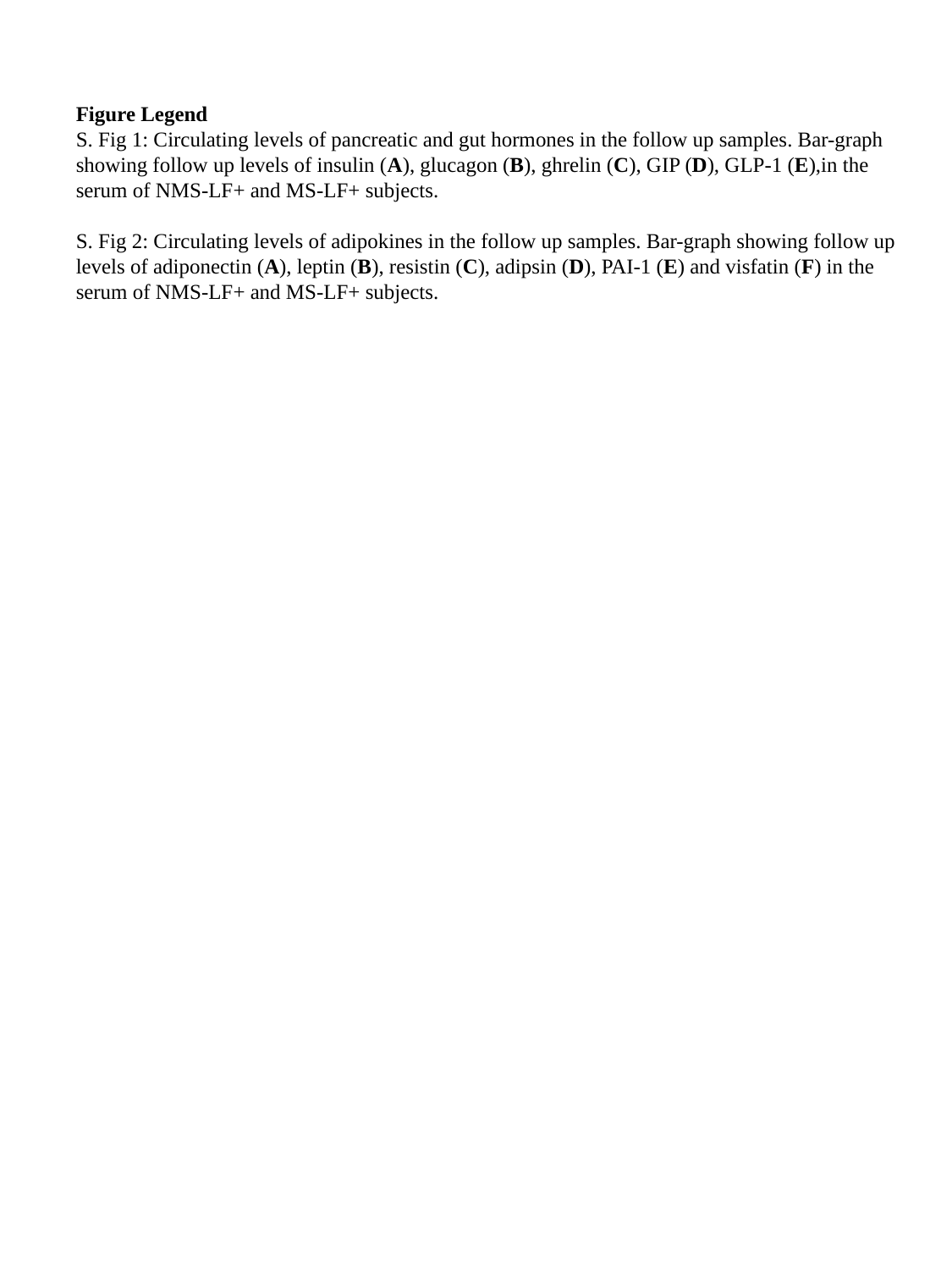

Figure Legend
S. Fig 1: Circulating levels of pancreatic and gut hormones in the follow up samples. Bar-graph showing follow up levels of insulin (A), glucagon (B), ghrelin (C), GIP (D), GLP-1 (E),in the serum of NMS-LF+ and MS-LF+ subjects.
S. Fig 2: Circulating levels of adipokines in the follow up samples. Bar-graph showing follow up levels of adiponectin (A), leptin (B), resistin (C), adipsin (D), PAI-1 (E) and visfatin (F) in the serum of NMS-LF+ and MS-LF+ subjects.

## Slide 2
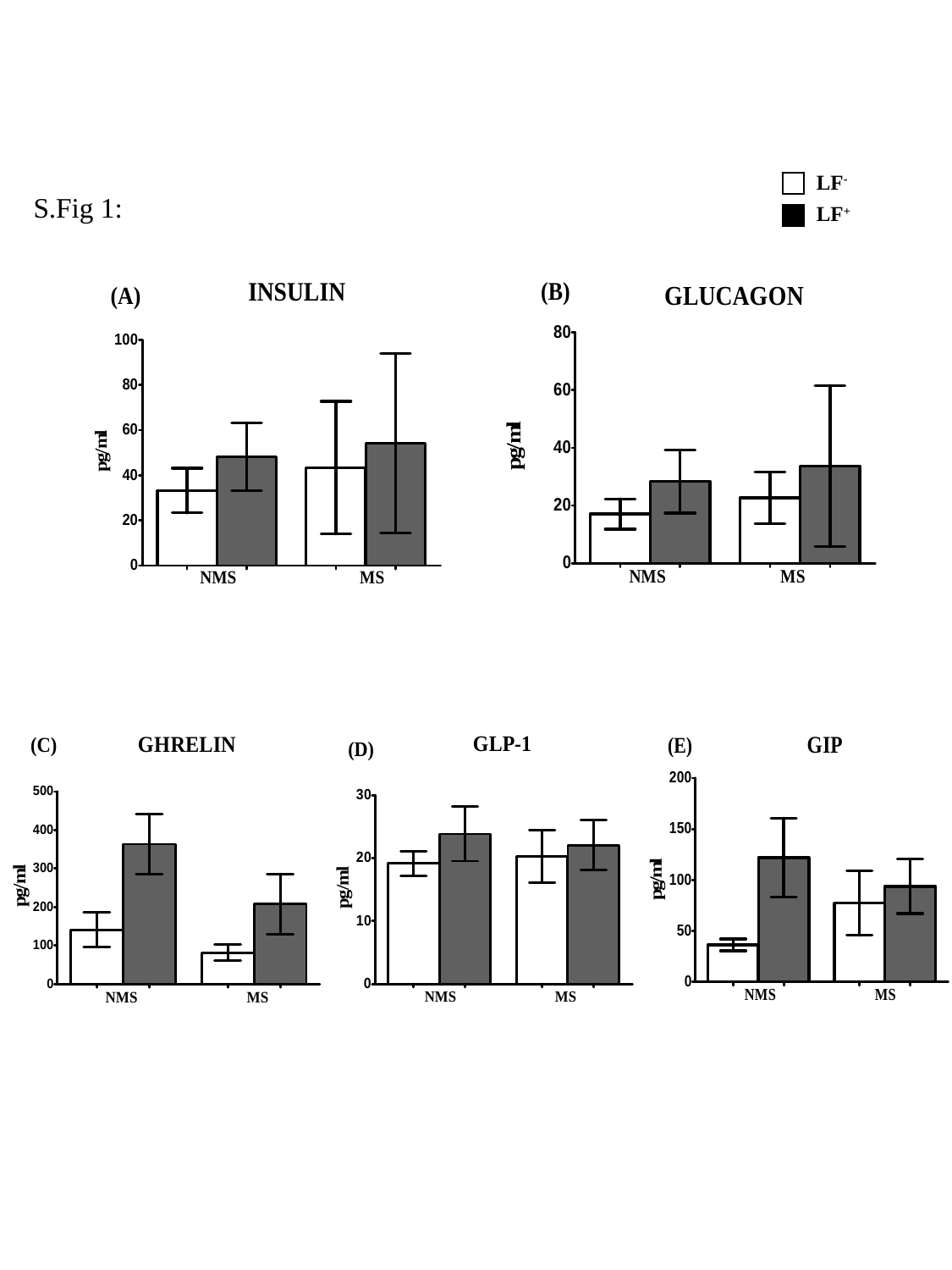

LF-
S.Fig 1:
LF+

## Slide 3
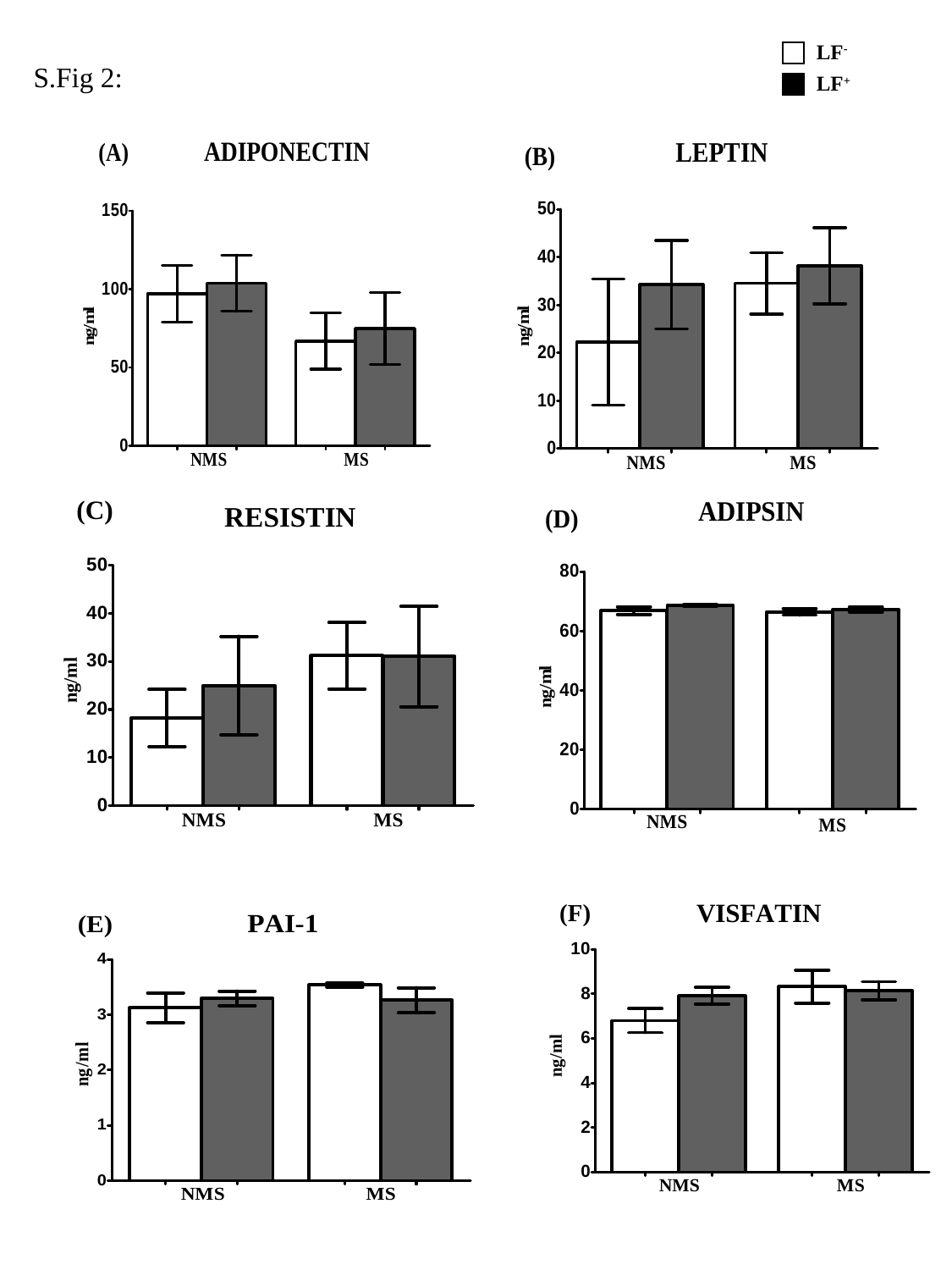

LF-
S.Fig 2:
LF+
